# Physician’s Prescription Book

**Published:** 1865-09

**Authors:** 


Physician's Prescription Book. Containing lists of the terms, phrases, contractions, and abreviations used in prescriptions, with explanatory notes; the grammatical construction of prescriptions; rules for the pronunciation of pharmaceutical terms; a prosodical vocabulary of the names of drugs, etc.; and a series of abbreviated prescriptions, illustrating the use of the preceding terms. To which is added a Key, containing the prescriptions in an unabbreviated form, with a literal translation. For the use of Medical and Pharmaceutical Students. By JONATHAN PERIRA, M.D., F.R.S. Fourteenth edition. Philadelphia: LINDSAY & BLAKISTON. 1865.

This is a small duodecimo volume, of near 300 pages, the contents of which are fully indicated by the title quoted above. It is a very convenient and useful work for reference; and the fact that it has reached the fourteenth edition, is sufficient evidence that its merits are appreciated by the Profession.

